# Supplementary material for: The effect of early oral postoperative feeding on the recovery of intestinal motility after gastrointestinal surgery: Protocol for a systematic review and meta-analysis
Source: PLoS One. 2022 Aug 18;17(8):e0273085. doi: 10.1371/journal.pone.0273085 (PMC9387793; doi:10.1371/journal.pone.0273085)
Supplement: S2 Appendix — (DOCX) [file pone.0273085.s002.docx]

Appendix 1. PUBMED search strategy

| #1 | exp Surgical Procedures, Operative/ OR xp Digestive System/ OR“upper abdominal surgery”.tw. OR“abdominal surgery”.tw. OR“gastrointestinal surgery”.tw. OR“surgical procedure”.tw. OR“reconstructive surgical procedures”.tw. OR  “digestive system surgical procedure”.tw. OR“[digestive system](https://www.cochranelibrary.com/advanced-search/mesh#0)”.tw. OR“upper gastrointestinal tract”.tw. OR “lower gastrointestinal tract”.tw. OR“gastrointestinal tract”.tw. OR gastric.tw. OR stomach.tw. ORepigastric.tw. OR  gastrectomy.tw. OR,“Billroth I”.tw. OR,“Billroth II”.tw. OR”“[Roux-en-Y](https://pubmed.ncbi.nlm.nih.gov/30117560/)”.tw. OR  “partial gastrectomy”.tw. OR,“total gastrectomy”.tw. OR“distal gastrectomy”.tw. ORgastropexy.tw. OR oesophagus.tw. OR esophagus.tw. OR esophageal.tw. OR  oesophageal.tw. OR esophagectomy.tw. OR oesophagectomy.tw. OR  esophagostomy.tw. OR oesophagostomy.tw. OR esophagotomy.tw. OR  oesophagotomy.tw. OR esophagoplasty.tw. OR oesophagoplasty.tw. OR  “esophagogastric junction”.tw. OR “oesophagogastric junction”.tw. OR  “esophago-gastric junction”.tw. OR “oesophago-gastric junction”.tw. OR  cardia.tw. OR hellermyotomy.tw. OR pylorus.tw. OR pyloric.tw. OR  [pyloromyotomy](https://www.ncbi.nlm.nih.gov/mesh/2023386).tw. OR pancreas.tw. OR pancreatic.tw. OR[pancreatectomy](https://www.cochranelibrary.com/advanced-search/mesh#0).tw. OR  [p](https://www.cochranelibrary.com/advanced-search/mesh#0)ancreaticoduodenectomy.tw. ORduodenopancreatectomy.tw. OR pancreatoduodenectomy.tw. OR [pancreaticojejunostomy](https://www.cochranelibrary.com/advanced-search/mesh#0).tw. OR pancreatojejunostomy.tw. ORbiliary.tw. OR“bile duct”.tw. OR liver.tw. OR hepatic.tw. OR hepato.tw. OR hepato-biliary.tw. OR biliopancreatic.tw. OR cholecist.tw. OR gallbladder.tw. OR“gall bladder”.tw. ORcholecystectomy.tw. OR  [cholecystostomy](https://www.cochranelibrary.com/advanced-search/mesh#0).tw. ORc[holedochostomy](https://www.cochranelibrary.com/advanced-search/mesh#0).tw. OR hepatectomy.tw. OR intestine.tw. OR intestinal.tw. OR colonic.tw. OR gut.tw. OR bowel.tw. OR ileum.tw. OR ileo.tw. OR  anal canal.tw. OR cecum.tw. OR colon.tw. OR rectum.tw. OR rectal.tw. OR colorectal.tw. OR sigmoid.tw. OR sigmoidal.tw. OR[jejunum](https://www.cochranelibrary.com/advanced-search/mesh#0).tw. OR colectomy.tw. OR sigmoidectomy.tw. OR proctocolectomy.tw. OR proctectomy.tw. ORsphincterotomy.tw. OR  diverticulectomy.tw.) |
| --- | --- |
| #2 | (exp Nutrition Therapy/ OR exp Diet, Food and Nutrition/ OR "early oral intake".tw. OR "early oral feeding".tw. OR "early enteral feeding".tw. OR "feeding methods".tw. OR  eating.tw. OR nutrition.tw. OR"nutrition therapy".tw. OR intake.tw. OR "late oral feeding".tw. OR "early feeding".tw. OR "early nutrition".tw. OR "enteral nutrition".tw. OR diet.tw. OR fasting.tw. OR "postoperative nutrition".tw. OR "sip feeding".tw. OR  "tube feeding".tw. OR nutritional.tw. OR "early enteral nutrition".tw. OR "dietary intake".tw. OR food.tw. OR liquid.tw. OR "clear liquid diet".tw. OR "typical diet".tw. OR  sip.tw. OR "oral intake".tw. OR "semi-solid".tw. OR semisolid.tw. OR semiliquid.tw. OR  "semi-liquid".tw. OR "nil by mouth".tw. OR "food avoidance".tw. OR "soft diet".tw. OR  "regular diet".tw. OR water.tw. OR “late enteral feeding”.tw. OR “late feeding”.tw. OR  “late nutrition”.tw. OR “late enteral nutrition”.tw. OR “late oral intake”.tw. OR  “enhanced recovery after surgery”.tw.) |
| #3 | (exp Signs and Symptoms, Digestive/ OR exp Digestive System Physiological Phenomena/ OR exp Ileus/ OR motility.tw. OR dysmotility.tw. OR nausea.tw. OR  cramp.tw. OR cramping.tw. OR pain.tw. OR ache.tw. OR "gastrointestinal distress".tw. OR spasm.tw. OR flatus.tw. OR "epigastric pain".tw. OR regurgitation.tw. OR  “feeding tolerance”.tw. OR “oral tolerance”.tw. OR "postoperative ileus".tw. OR "gut motility".tw. OR “gut dysmotility”.tw. OR "bowel dysfunction".tw. OR  “bowel function”.tw. OR "intestinal dysfunction".tw. OR “intestinal function”.tw. OR  "gastrointestinal motility".tw. OR "gastrointestinal dysmotility".tw. OR "gastrointestinal symptoms".tw. OR "gastrointestinal function".tw. OR "gastrointestinal dysfunction".tw. OR  "gastrointestinal distress".tw. OR "gastrointestinal disturbance".tw. OR "gastrointestinal adverse events".tw. OR "gastrointestinal transit".tw. OR "gastrointestinal disorders".tw. OR  "abdominal discomfort".tw. OR "abdominal comfort".tw. OR "abdominal pain".tw. OR  "abdominal distension".tw. OR "abdominal dysfunction".tw. OR  "gastric motility".tw. OR “gastric dysmotility”.tw. OR constipation.tw. OR  defecation.tw. OR dyspepsia.tw. OR stool.tw. OR vomiting.tw. OR "paralytic ileus".tw. OR  ileus.tw. OR bloating.tw. OR “bowel function”.tw. OR “intestinal function”.tw.) |
|  | #1 and#2 and #3 |

**Filter**

Clinical Trial, Controlled Clinical Trial, Randomized Controlled Trial

English, German, Italian

Adult: 19+ years

from 1990 to 2021

Title/Abstract
