# Supplementary material for: The effect of early oral postoperative feeding on the recovery of intestinal motility after gastrointestinal surgery: Protocol for a systematic review and meta-analysis
Source: PLoS One. 2022 Aug 18;17(8):e0273085. doi: 10.1371/journal.pone.0273085 (PMC9387793; doi:10.1371/journal.pone.0273085)
Supplement: Attachment — Submitted filename: Response to Reviewers-2.docx [file pone.0273085.s003.docx]

**Response to reviewers**

Dear Reviewers,

thank you for your feedback.

We have really appreciated your suggestions.

Each point has been considered with care and appropriately addressed as indicated below.

We have introduced the changes required and we have highlighted them with Track Changes.

Thank you again for your attention and consideration.

**Reviewer #1: I would recoomend a program like Covidence for systematic review management, with proper blinding of the reviewers**

Thank you for your feedback. Me and my co-authors have discussed it and we have decided to move forward with this suggestion. We added this information in the Data collection and analysis section.

**Reviewer #2: Thank you for the opportunity to review this manuscript - I have found the topic interesting and important to the field of gastrointestinal surgery. A protocol for a systematic review and meta-analysis of the impact of the effect of early oral postoperative feeding on the recovery of intestinal motility.**

**The authors tackle a major challenge in gastrointestinal surgery. They correctly identify the current literature, with the most up-to-date systematic review results including data on the effect of early post-operative feeding. The methodology for the review is sound with minor comments below.**

**My only limitation lies with what this review will add to the literature, given recent relevant systematic reviews that have covered this topic.**

This is a very interesting point. The reserch group is aware that there are other recent SRs on this topic but limited to colorectal or lower gastrointestinal surgical patients. Moreover, the outcomes were heterogeneus and other primary outcomes rather than post-operative ileus were measured. We added these reasons in the “Why it is important to do this review”section.

**Please consider the following comments.**

**1. Please could the authors explain their reasoning for language and year restriction in line with AMSTAR 2 guidance. Also, an explanation for non-randomised trial exclusion is warranted.**

We added the information requested in the Types of studies paragraph.

**2. I appreciate the potential reasons for why bariatric surgery was excluded, however the protocol would benefit from a transparent explanation to the reader.**

Thank you for rising this relevant point. We deepen discussed on this issue whitin the reserch group during the protocol development. Thus, we decided to exclude the bariatric surgery due to its metabolic nature that implies different kind of effects and a post-operative care tailored to patient’s needs (PMID: 24194467). We added a statement in the “Types of participants” paragraph.
